# Authors' response (August 19, 2026) to the Letter to the Editor concerning the paper “Factors associated with respiratory symptoms in communities living near to and away from the railway used for coal transportation in La Guajira, Colombia”

**DOI:** 10.13075/ijomeh.1896.02978

**Published:** 2026

**Authors:** Jeannette Liliana Amaya-Lara, Jesús Rodríguez-García, Rolando Enrique Peñaloza-Quintero, Marino Mauricio Mejía-Rocha

**Affiliations:** Pontificia Universidad Javeriana, Institute of Public Health, Bogotá, Colombia

**Keywords:** risk factors, air quality, respiratory symptoms, Colombia, coal mining, trains

Dear Editor,

We sincerely appreciate the interest shown by the author of the Letter to the Editor [1] regarding our article [2] and welcome the opportunity to clarify several aspects of our study. We value this discussion because it provides an opportunity to further explain the rationale underlying our methodological decisions and the interpretation of our findings.

We fully acknowledge that coal mining is an important environmental determinant in La Guajira and that its potential effects on environmental quality and human health deserve continued scientific investigation. We also recognize that the region has a complex socio-environmental context that is highly relevant to environmental health research. However, the objective of our study was not to evaluate environmental conflicts or attribute responsibility to a single source of emissions. Rather, our aim was to identify factors associated with respiratory symptoms in communities living near and away from the railway used for coal transportation through an epidemiological approach. These objectives are complementary but methodologically distinct.

From a public health perspective, respiratory health results from the interaction of multiple environmental and individual determinants. In the study area, exposure to airborne particulate matter may arise from coal extraction and transportation, but also from unpaved roads, vehicular traffic, biomass burning, household combustion, wind-driven resuspension of soil, climatic conditions, and natural desert dust. Considering these multiple determinants was not intended to minimize the potential contribution of mining activities but to better characterize the complex environmental exposures experienced by the population. Such an approach is consistent with the principles of environmental epidemiology and supports the development of integrated public health interventions.

To further clarify the context of the study, we also wish to note that the study was indeed funded by Carbones del Cerrejón Limited (contract No. 21682016, research entitled “Association between the respiratory health of communities and proximity to the Cerrejón mining and port operation, taking into account other risk factors,” manager: Rolando Enrique Peñaloza-Quintero). The sponsor had no role in the study design, data collection, data analysis, interpretation of the results, manuscript preparation, or the decision to submit the manuscript for publication. The authors retained full scientific independence throughout the entire research and publication process.

Regarding the concern about possible overadjustment, we respectfully clarify that the outcome analyzed in our study did not correspond to physician-diagnosed asthma, pneumonia, bronchitis, or chronic obstructive pulmonary disease. Instead, the outcome was defined through a symptom-based algorithm designed to identify individuals presenting respiratory symptom patterns, including persistent cough with sputum, wheezing associated with dyspnea, and other respiratory warning signs. Conversely, the explanatory variables questioned by the author represented physician-diagnosed conditions for which participants reported having received treatment during the previous year. Although these variables are clinically related to respiratory symptoms, they are conceptually different from the symptom-based outcome and were incorporated to account for differences in participants' underlying health status that could influence the occurrence of respiratory symptoms. We acknowledge that alternative model specifications may also be considered, and we appreciate the opportunity to discuss these methodological choices.

With respect to the use of odds ratios, the pooled logistic regression model was selected because the outcome was binary and repeatedly measured across 3 study visits, allowing estimation of adjusted associations while accounting for multiple covariates. The odds ratio is the natural measure of association derived from this model. We recognize that when an outcome is relatively common, odds ratios should not be interpreted as equivalent to risk ratios and may differ in magnitude. Accordingly, the estimates reported in our study should be interpreted as adjusted odds ratios rather than as relative risks. This consideration concerns the interpretation of the effect measure, but it does not invalidate the analytical approach or, by itself, explain the absence of statistically significant associations between particulate matter concentrations or residential proximity to the railway and respiratory symptoms.

Finally, we respectfully believe that our findings should not be interpreted as minimizing the potential environmental health impacts of coal transportation. Our article does not conclude that coal transportation has no effect on respiratory health. Rather, it reports that no statistically significant association was identified under the conditions of this study while explicitly acknowledging several important methodological limitations that may have influenced these findings. These limitations included the spatial distribution of air-monitoring stations, the inability to distinguish the specific sources of particulate matter, the absence of indoor air quality measurements, the lack of information on wind direction, and the use of symptom-based rather than clinically confirmed respiratory outcomes. We therefore believe that our conclusions appropriately reflect the scope of the available evidence and the limitations of the study, and should not be interpreted as evidence of the absence of environmental risk.

We agree that research conducted in regions affected by complex environmental exposures should continue to integrate rigorous epidemiological methods, improved exposure assessment, environmental monitoring, and dialogue with affected communities. We hope that our study contributes to this ongoing effort and encourages future investigations that further strengthen the evidence on environmental determinants of respiratory health in La Guajira.

We thank the author once again for contributing to this important scientific discussion.
